# A case report and anesthetic implications of situs inversus totalis in video-assisted thoracoscopic pulmonary resection

**DOI:** 10.1097/MS9.0000000000001895

**Published:** 2024-03-05

**Authors:** Benjamin T. Kor, Jennifer J. Kor, Todd M. Kor, Amy Y. He, Thomas M. Stewart, Charles R. Sims, Ryan E. Hofer, Nathan T. Kor

**Affiliations:** a Department of Anesthesiology and Perioperative Medicine, Mayo Clinic, Rochester, Minnesota; b Kentucky College of Osteopathic Medicine, University of Pikeville, Pikeville, Kentucky, USA

**Keywords:** anesthetic management, lung malignancy, SIT, situs inversus totalis, thoracoscopy

## Abstract

**Introduction and importance:**

Reports are limited on video-assisted thoracoscopic surgery for lung malignancy of patients with situs inversus totalis (SIT). Patients with SIT have significant anatomic differences with implications that are important for surgery, anesthesia, and nursing to understand in order to provide care for this patient population.

**Case presentation:**

A 64-year-old man with SIT and lung adenocarcinoma needed flexible bronchoscopy and wedge resection of a 9×8 mm adenocarcinoma in the right upper lobe and underwent video-assisted thoracoscopic surgery.

**Clinical discussion:**

Preoperative planning, including collaboration with the surgical team, allowed safe monitoring, induction of anesthesia, and airway isolation in this patient allowing them to have successful resection of their pulmonary malignancy. Postoperative care was enhanced by detailed communication and understanding of the patient’s anatomy and implications of this condition for post anesthesia care unit nursing care.

**Conclusion:**

Patients with rare clinical conditions and backgrounds may require surgical and anesthetic intervention. The authors describe important anesthetic considerations of preoperative evaluation, airway management, cardiac monitoring, and vascular access that should be noted and taken into account for patients with SIT. Proper preparation, planning, and communication allow for patients with SIT to safely undergo surgical procedures.

## Introduction

Highlights

- Situs inversus totalis (SIT) is a rare genetic condition.
- Thoracic malignancy in patients with SIT present unique anesthetic considerations.
- Airway management in patients with SIT may be challenging.
- Communication between perioperative teams is critical to ensure safe care of patients with SIT.
- Patients with SIT may safely undergo surgery if their care teams understand the clinical implications of their condition.

Situs solitus refers to normal positioning and morphologic characteristics of thoracic and abdominal organs on their expected sides of the body. Levocardia refers to positioning of the heart with the apex of the heart in the left side of the thorax. Anomalies in embryologic development may result in alterations to this typical anatomical location of internal organs. Situs inversus totalis (SIT), dextrocardia, and situs ambiguous are congenital alterations to the expected anatomical locations of the affected organs.

SIT is a congenital condition in which major internal organs are transposed in a mirrored orientation along a sagittal plane relative to normal anatomy^1–3^. It occurs in 1 to 2 per 10 000 persons^4^. SIT is thought to occur as a result of an X-linked autosomal recessive genetic transmission^5^. Intrathoracic pulmonary and cardiac anatomy consists of three left and two right lung lobes and dextrocardia, or location of the apex of the heart into the right side of the chest. Intra-abdominal organs are transposed similarly, with the liver, spleen, and appendix positioned in the left side of the abdominal cavity. Most patients with SIT do not have additional congenital anomalies; however, the frequency of congenital heart disease has a small increase, with transposition of the great vessels occurring with the greatest frequency^4,6,7^. Most patients with SIT have no medical symptoms and receive the diagnosis on routine examination of heart sounds or after evaluation for conditions unrelated to SIT.

Patients with SIT who require thoracic surgery have distinct perioperative challenges for surgical and anesthesia teams. The literature is sparse for the description of lung malignancy and perioperative management for patients with SIT^8,9^.

A written Health Insurance Portability and Accountability Act authorization to use/disclose existing protected health information was obtained from the patient for publication of this case. This case report was published with the written consent of the patient.

This case report has been reported following the Surgical CAse REport (SCARE) guidelines^10^.

## Case presentation

A 64-year-old man with adenocarcinoma of the lung presented for flexible bronchoscopy and wedge resection of a 9×8 mm adenocarcinoma in the right upper lobe. The procedure was performed via a video-assisted thoracoscopic approach.

**Figure 1:**
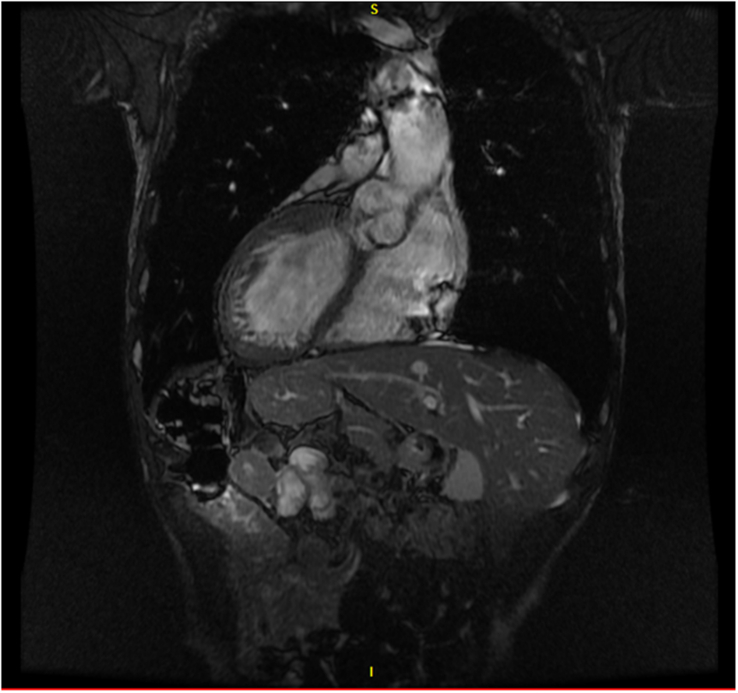
Computed tomography image of patient with situs inversus totalis. Of note, the image shows dextrocardia and mirror imaging of thoracic and abdominal contents.

The patient had received the diagnosis of SIT (Fig. 1) as a child. Other pertinent past medical history included hypothyroidism, extensive smoking with a greater than 84 pack year history, moderate chronic obstructive pulmonary disease, and stable mid ascending aortic dilation measuring 45 mm. Serial radiographic evaluation of his aorta led to the incidental finding of a right upper lobe pulmonary nodule. The patient underwent computed tomography (CT)-guided biopsy of the pulmonary lesion, which confirmed the diagnosis of adenocarcinoma. Additionally, the patient had a PET-CT scan, pulmonary function testing showing a FEV1 of 1.57 L and a DLCO of 49% predicted. Imaging, including chest and brain MRI, did not find any evidence of mediastinal or hilar adenopathy nor any evidence of metastatic disease. Review of the patient’s history, examination, imaging, and multidisciplinary evaluations led the surgical team to determine the best course for surgical excision to be thoracoscopic wedge resection of the patient’s lesion to allow the patient to maintain as much of his lung as possible while ensuring clear margins during resection. Intraoperative pathologic tissue examination revealed clear margins and complete resection of the lesion. The patient denied a history of chronic sinusitis, bronchiectasis, hearing loss, infertility, or other signs of primary ciliary dyskinesia or Kartagener syndrome. The latter is associated with SIT in up to 25% of cases^11,12^.

The patient underwent CT-guided technetium Tc 99 nodule localization before operative intervention. On arrival in the operating room, the patient had standard American Society of Anesthesiologists (ASA) monitors applied. Because of the diagnosis of SIT, ECG leads were placed in a mirrored orientation on opposite sides of the patient’s chest to allow monitoring of typical electrical orientation of the heart. The LA (left arm), LL (left leg), and V (chest) leads were placed on the patient’s right chest, and the RA (right arm) and RL (right leg) leads were placed on his left chest. After an uneventful induction of general anesthesia, his airway was secured with direct laryngoscopy and placement of a single-lumen endotracheal tube (ETT).

Flexible fiberoptic video bronchoscopy anatomy was consistent with CT findings and the patient’s history of SIT. The patient’s anatomical left lung consisted of three lobes and the right lung of two lobes in a mirrored image of normal anatomy. The patient’s right lung (morphologic left) had a long mainstem bronchus divided into upper and lower segments and his left lung (morphologic right) had a proximal upper lobe bronchus and bronchus intermedius. Other than showing the mirrored orientation, the videobronchoscopic examination was unremarkable.

**Figure 2:**
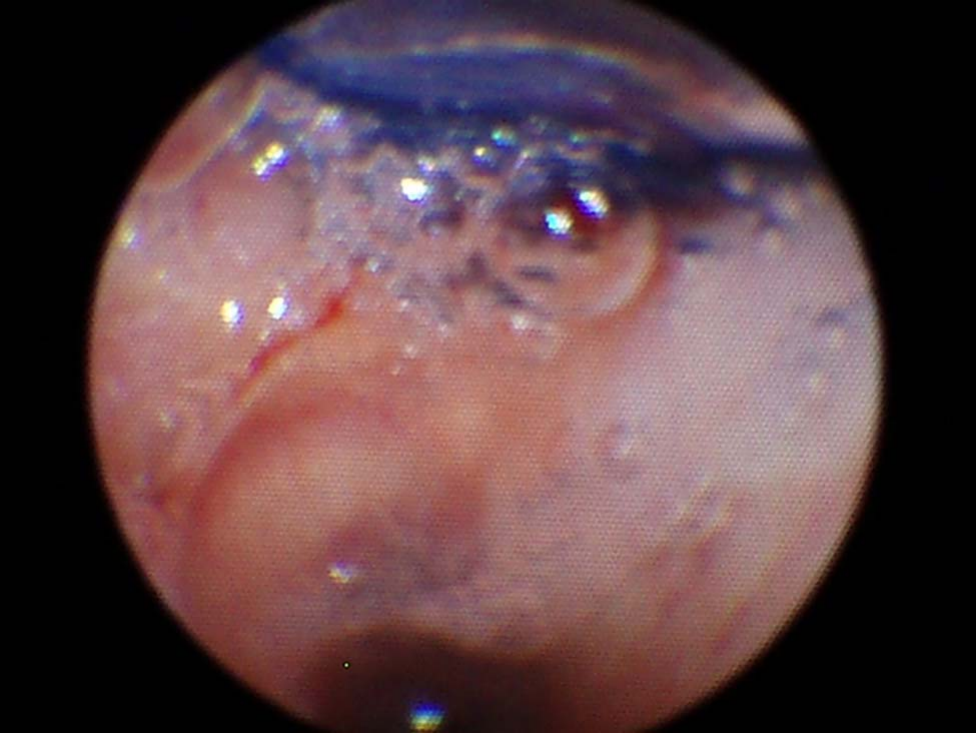
Bronchoscopic image confirming placement of a double lumen endotracheal tube in the right mainstem bronchus.

The patient’s airway and oropharynx were then suctioned and the single-lumen ETT removed. A left-sided double-lumen ETT (DLETT) was placed into his trachea under direct laryngoscopy and rotated 90° clockwise to advance the device into the patient’s right mainstem bronchus. Placement of a left-sided DLETT into the right mainstem bronchus for thoracic operation of this patient population has been described previously^13,14^. Flexible fiberoptic videobronchoscopy confirmed proper positioning of the DLETT with the bronchial balloon positioned immediately distal to the tracheal carina (Fig. 2). Bronchoscopy also confirmed patency of the patient’s right (functionally left) upper and lower lobes. The patient was then turned to a left lateral decubitus position, with all pressure points padded and an axillary roll placed. Repeat bronchoscopy confirmed the appropriate DLETT position. The right (functionally left) lung was then isolated, and ventilation to this lung was discontinued. Three 10 mm ports were used for the video-assisted thoracoscopic surgery (VATS) with radionucleotide-guided wedge resection of the lesion. The patient underwent wedge resection of the right upper lobe with use of an Endo GIA stapler (Medtronic). Pathologic evaluation of the tissue confirmed an adenocarcinoma with clear margins. A chest tube was placed, and several intercostal nerve blocks with liposomal bupivacaine were administered through video thoracoscopic guidance. A postoperative chest radiograph confirmed bilateral postoperative lung reinflation.

The patient had an unremarkable intraoperative and postoperative course and was discharged without complications on postoperative day 1. Follow up with the patient at 22 months postoperative revealed no evidence of recurrence of his malignancy.

## Clinical discussion

This case report presents an uneventful perioperative course for an uncommon clinical presentation, highlighting the important considerations of preoperative evaluation, airway management, cardiac monitoring, and vascular access that should be noted and taken into account for patients with SIT.

### Preoperative evaluation

Preoperative anesthetic considerations include a thorough history, physical examination, and imaging studies for evaluation for additional congenital comorbidities and a thorough understanding of the patient’s anatomy. SIT may be associated with other medical conditions, such as Kartagener syndrome, cardiac anomalies, mucociliary dysfunction, airway anomalies, and spleen malformations. It must be distinguished from conditions such as dextrocardia during the preanesthetic evaluation. Because of the dextrocardia associated with SIT, heart sounds should be auscultated in the right side of the patient’s chest.

### Airway management

Patients undergoing VATS require lung isolation to provide optimal lung visualization and a quiet surgical field. Options for lung isolation in intrathoracic surgery include placement of a left- or right-sided DLETT, a single-lumen ETT with a bronchial blocker, or advancement of a single-lumen ETT into the mainstem bronchus of the lung to be ventilated during the operation. In SIT, right lung anatomy would not have a short mainstem bronchus with a takeoff to the right upper lobe and a bronchus intermedius. Instead, the right lung would have an anatomically longer mainstem bronchus, as found in a morphologically normal left lung. For patients with SIT requiring lung isolation, the airway management of choice is a left-sided DLETT rotated 90° clockwise to final placement in the right mainstem bronchus. A right-sided DLETT rotated 90° counterclockwise to a final placement in the left mainstem bronchus could be used in rare situations, such as when a right bronchial sleeve resection is planned or cross-contamination of the tracheobronchial tree from the left lung is a concern.

### Cardiac monitoring and vascular access considerations

For a patient with SIT, electrocardiographic monitoring should have the leads reversed to monitor or document the patient’s cardiac electrical activity. If cardioversion or defibrillation is required, placement of defibrillator leads over the right side of the chest would be required. Peripheral vascular access should not be affected by the presence of SIT. However, when a central venous catheter is deemed to be appropriate, the left internal jugular vein should be considered because of the relation of the left internal jugular vein to the superior vena cava and left atrium for patients with SIT.

### Transfer of care

Detailed explanation of the medical condition should be conveyed to the surgical team during any intraoperative and postoperative transfer of care. Airway management such as for this patient results in an atypical extraoral DLETT orientation that can lead to confusion and inappropriate intervention if DLETT manipulation and repositioning are required.

## Conclusion

With the surgical team’s proper preparation, planning, and understanding of the unique differences from normal anatomy, patients with SIT may safely undergo thoracic surgery for intrathoracic pathologic conditions. Effective communication between the surgical team, anesthesiology and the postoperative care unit allowed for safe care of this patient by educating and informing the care teams of the unique anatomic and physiologic characteristics of our patient and its impact on monitoring and physical examination of this patient.

## Ethical approval

Patient consent for use of information and images was obtained from the patient. Additional IRB or Ethical approval for this case report was not required by our institution as the patient provided a signed consent authorizing use of patient information. We are reporting on the clinical care of a patient and not on any study or investigation of this patient or patient population.

## Consent

Written informed consent was obtained from the patient for publication and any accompanying

images. A copy of the written consent is available for review by the Editor-in-Chief of this journal on request.

## Sources of funding

No funding was provided.

## Author contribution

All authors contributed equally in creating the manuscript, editing, and approval of final manuscript.

## Conflicts of interest disclosures

No potential conflicts of interest relevant to this article was reported.

## Research registration unique identifying number (UIN)

The manuscript is a case report and brief review. This is not a research study or manuscript.

## Guarantor

Todd Kor.

## Data availability statement

Data sharing is not applicable.

## Provenance and peer review

Not commissioned, externally peer-reviewed.
